# Literary Note

**Published:** 1884-11

**Authors:** 


### LITERARY NOTE.

Messrs. Jansen, McClurg & Co., Chicago, have issued a new work, on the "Principles and Practice of Medicine," by Dr. N. S. Davis. The work is not a compilation, but an embodiment of the observations, thoughts, and experi. ences of the author during nearly fifty years of active medical practice. The matter is presented in the form of lectures, delivered by him during his many years of teaching. The features which especially commend the work to the practitioner and student, are the fullness with which the clinical history of the various diseases is given, and the explicit and detailed description of the methods of treatment which have been found most effective. The author's adoption of the metric system of weights and measures is worthy of notice and commendation. Although this system has been advocated by leading scientific and medical societies, it has come into use only to a limited extent. To assist in effecting this change, Dr. Davis has used the metric system throughout the work—giving, however, in brackets, the equivalents in apothecaries' measure.

The volume is about the size of Bartholow's "Practice of Medicine," but more closely printed. The author is well known throughout the country as one of the ablest and most original thinkers in the profession, who has won a high reputation as a lecturer upon practical medicine; and the profession is to be congratulated upon having, in a permanent form, the rich results of his busy professional life.

